# Intelligent microstructure materials for diagnosis and treatment of osteoarthritis: progress and AI-enpowered future

**DOI:** 10.1038/s41413-025-00458-5

**Published:** 2025-10-15

**Authors:** Weijin Gao, Jiahui Zhong, Xinyi Liu, Dan Bai, Mengjie Wu

**Affiliations:** 1 Stomatology Hospital, School of Stomatology, Zhejiang University School of Medicine, Zhejiang Provincial Clinical Research Center for Oral Diseases, Hangzhou, China; 2 Key Laboratory of Oral Biomedical Research of Zhejiang Province, Cancer Center of Zhejiang University, Engineering Research Center of Oral Biomaterials and Devices of Zhejiang Province, Hangzhou, China; 3 Pharmaceutical Informatics Institute, College of Pharmaceutical Sciences, Zhejiang University, Hangzhou, China

**Keywords:** Bone quality and biomechanics, Bone

## Abstract

Osteoarthritis (OA) is a widespread joint disorder that has emerged as a significant global healthcare challenge. Over the past decade, advancements in material science and medicine have transformed the development of functional materials aimed at addressing the complex issues associated with the diagnosis and treatment of OA. This review synthesizes the latest advancements in various types of intelligent micro-structured materials and their design principles. By examining the exceptional structural characteristics of materials with unique properties such as tailored attributes, controllability, biocompatibility, and bioactivity, we emphasize the design of composite materials for precise and early intervention in OA. This is achieved through advanced imaging techniques and machine learning-based analysis, alongside the customization of micro-structured material properties to align with the biological and mechanical requirements of specific joint tissues. This review offers an in-depth analysis of the transformative potential of advanced technologies and artificial intelligence (AI) in the development of innovative solutions for OA diagnosis and therapy. It aims to inform future research and inspire the creation of next-generation smart materials with unprecedented performance, thereby enhancing our capabilities in the prevention and treatment of OA.

## Introduction

Osteoarthritis (OA) is a prevalent degenerative disorder that frequently results in joint pain and restricted mobility, affecting over 500 million individuals worldwide and imposing considerable physical, psychological, and economic burdens on those afflicted.^1^ The management of OA is notably challenging due to the limited self-repair capacity of articular cartilage and the absence of specific diagnostic biomarkers, which hinder both therapeutic and diagnostic approaches.^2^ Consequently, there is a critical need to explore effective strategies for the early diagnosis and precise treatment of OA through the development and application of innovative functional materials.

**Fig. 1:**
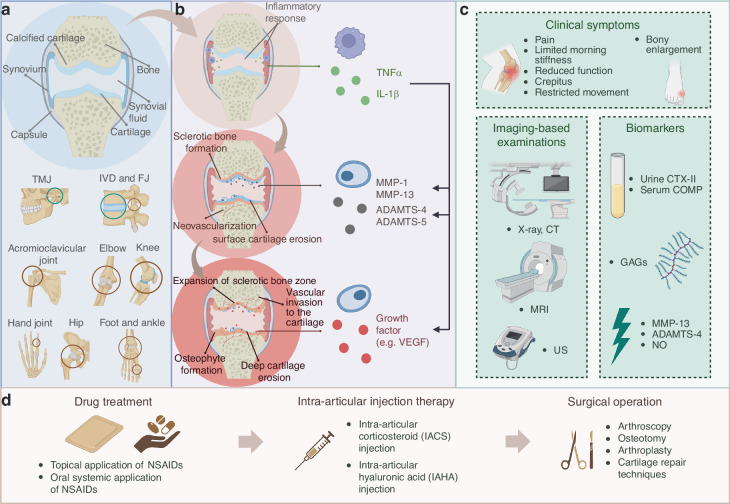
Typical structures of healthy joints, pathological progress, diagnostic methods and therapeutic strategies of OA. **a** The general structure of joints and the susceptible sites (TMJ Temporomandibular Joint, IVD Intervertebral Disc, FJ Facet Joint). The joints framed by green circles are mainly covered by fibrocartilage on the articular surface, while those in brown circles are mainly covered by hyaline cartilage. **b** Pathological changes in the progression of OA. **c** Existing clinical diagnosis methods for OA diagnosis. **d** Current clinical strategy for OA therapy. Created with BioRender.com

The management of OA confronts multiple challenges: (1) the multifactorial and heterogeneous etiology, which includes factors such as trauma, aging, mechanical overloading, and obesity;^3^ (2) the limited self-repair capacity of articular cartilage and the lack of specific diagnostic biomarkers;^2^ and (3) the unique biomechanical compatibility requirements necessitated by anatomical variations across joints (Fig. 1a) for diagnostic and therapeutic materials.^4,5^ At the molecular level, the complex pathological features (Fig. 1b), including synovial inflammation infiltrated by cytokines, cartilage degradation facilitated by matrix-degrading enzymes, and aberrant vascular and neural hyperplasia mediated by growth factors, further exacerbate the challenges associated with the treatment and diagnosis of OA.^6–8^

Current diagnostic methodologies (Fig. 1c) predominantly rely on clinical manifestations and imaging techniques to identify irreversible structural changes within the joint.^9–11^ According to clinical practice guidelines, contemporary treatment recommendations for OA, such as intra-articular injection therapy, are strongly advocated.^12–14^ However, these clinical interventions exhibit significant limitations, including inadequate specificity, suboptimal drug delivery, and adverse effects.^15^ Consequently, there is an urgent need for the development of more advanced and precise tools to facilitate the effective and safe diagnosis and treatment of OA.

**Fig. 2:**
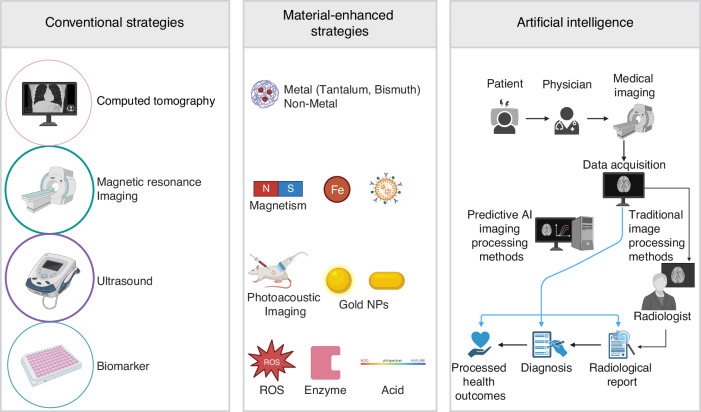
Schematic illustration of the conventional, material-enhanced and AI-enpowered strategies for OA diagnosis. Created with BioRender.com

Initially, biomaterials were regarded as passive structural components or simple pharmaceutical carriers. The proposal of the conception “smart biomaterials” has revolutionized this paradigm by introducing dynamic responsiveness and molecular recognition capabilities. Since then, the concept has been continuously expanded, evolving from macroscopic structures to micro-level systems (see Fig. S1).^16,17^ In 2021, Montoya et al. proposed a classification system for smart biomaterials based on their level of interaction with the microenvironment, particularly in relation to biological and cellular processes.^18^ Additionally, they demonstrate enhanced therapeutic precision through three key innovations: 1) stimuli-responsive drug release mechanisms activated by pathological biomarkers, 2) targeted delivery systems utilizing ligand-receptor interactions, and 3) self-regulating matrices that adapt to microenvironmental changes.

Though both conventional and smart materials fundamentally rely on their inherent physicochemical and biological properties to perform functions, smart materials are characterized by their ability to exert targeting effects, or to trigger cellular and tissue responses. These distinctions make the diagnosis and treatment more controllable and visual.^17–20^ Particularly in OA management, where complex pathological mechanisms involving inflammatory cascades and cartilage degradation coexist, these intelligent platforms enable more precise spatiotemporal control over therapeutic agent release and disease-specific targeting compared to traditional delivery approaches. Materials such as nanoprobes targeting personalized biomarkers enable early diagnosis prior to joint damage, while stimuli-responsive systems using external stimuli (light, mechanical force, magnetism, ultrasound) and endogenous OA indicators (pH and temperature changes, reactive oxygen species [ROS] levels) optimize therapeutic delivery through material-mediated delivery systems, including nanoparticles (NPs) and hydrogels.^21–23^ With the integration of artificial intelligence (AI), these distinct features now extend to personalized precision and AI-driven structural standardization/optimization of biomaterials.^24,25^ Furthermore, advancements in AI and synthetic techniques have led to the development of smart materials that are increasingly subtle, functional, and precise.^26,27^

This review offers a thorough and systematic examination of intelligent microstructure biomaterials for the diagnosis and treatment of OA. It provides a foundational overview of smart materials, highlighting recent advancements in their design, fabrication, and modification. Additionally, the transformative role of AI in enhancing diagnostic prognostication, material design, and optimization is investigated, aiming to bridge the gap between research and clinical applications. Significant attention is devoted to advanced fabrication techniques, such as additive manufacturing (3D and 4D printing) and microfluidics, which facilitate precise control over composition and architecture. These innovations are pivotal in driving the development of next-generation, precision-engineered smart materials for the management of OA. When combined with AI technology, these strategies—already enhanced by smart materials—can be significantly improved to achieve more precise OA diagnosis (Fig. 2).

## Smart materials for OA diagnosis

Prompt and precise diagnosis of OA is essential for effective disease management and improved patient outcomes. Clinically, OA diagnosis is primarily based on the patient’s reported symptoms and physical examination findings. Radiological imaging serves as a valuable adjunct for early diagnosis and the exclusion of other pathologies. X-ray imaging is capable of detecting joint space narrowing and bone spurs. Although it is cost-effective and easily accessible, its sensitivity is relatively low, thus limiting its effectiveness.^28^ Computed tomography (CT) provides enhanced visualization of mineralized tissues, facilitating a more effective assessment of bone mineral density.^29^ Magnetic resonance imaging (MRI) surpasses radiography in sensitivity, particularly in identifying soft tissue changes and early pathological alterations with high-resolution detail.^30^ Ultrasound imaging, known for its high sensitivity and specificity, can reveal joint effusion, bone spurs, and other features, although its accuracy is heavily reliant on the operator’s expertise.^31^ Nevertheless, by the time clinical symptoms are apparent, the disease often has progressed to an advanced stage, and existing imaging modalities have inherent limitations. Consequently, there is a pressing need for the development of novel diagnostic tools aimed at early detection.

Recent advancements in smart materials, particularly the development of microstructure materials, have revolutionized the landscape of OA diagnosis, making the diagnosis procedure smarter and more precise. NPs-based contrast agents improve in vivo detection and enhance targeting efficiency of CT. The use of metal NPs with different surface modifications allows MRI contrast agents to have better targeting accuracy, higher affinity, and improved imaging quality. Novel NPs coated with different bioactive substances improve the thermal conversion efficiency of photoacoustic imaging (PAI). With individual strategies (Table 1), materials designed to target different biomarkers increase the sensitivity of the diagnosis, leading to the reduction of the reliance on the clinician’s subjective experience for diagnosis.

**Table 1:** Classification of smart materials for OA diagnosis

Means of diagnosis	Smart strategies	Smart materials	Intelligent response
CECT	Intrinsic property	Ta_2_O_5_	Positively charged NPs exhibit enhanced affinity for articular cartilage.
	Intrinsic property	TaO	The CECT attenuation of cationic and neutral NPs exhibited a correlation with both GAG content and equilibrium modulus.
	Intrinsic property	Ioxaglate, Bi_2_O_3_	Diffusivity discrepancy between Ioxaglate and Bi₂O₃.
	Intrinsic property	CPC, mAv	The mixture can penetrate the full thickness of cartilage through electrostatic interactions and elicit a CT signal.
MRI	Peptide targeting	ALN-SPIONs	Bone-targeting NPs selectively bind to hydroxyapatite matrices in bone tissue.
	Peptide targeting	SPIONs, WYRGRL	Peptide-modified NPs specifically target type II collagen in cartilage matrix.
	Peptide targeting	Gd_2_(CO_3_)_3_, GdPDW	High cartilage affinity and MR imaging compatibility.
	Peptide targeting	WY-CMC-MnO	Early lesion-targeting NPs with improved MR imaging resolution.
PAI	Peptide targeting	Au@PDA-WL	Collagen II-targeting NPs aggregate on cartilage surface with high thermal conversion efficiency.
	Specific recognition	MoS_2_-AuNR, NGF antibodies	NGF-targeting probes identify pain-associated OA knees.
	Specific recognition	PLL-MNPs	Anionic GAG activity modulates PA signal intensity (melanin-dependent).
Biomarker	Enzymatic reaction	CMFn	CMFn emits light for OA imaging in response to MMP-13 overexpression.
	Enzymatic reaction	MRC-PPL@PSO	MMP-13 cleaves micelles to release fluorescent dyes in OA microenvironment.
	Enzymatic reaction	ERMs@siM13	ERMs@siM13 releases fluorescent dye upon MMP-13 cleavage.
	Enzymatic reaction	ADAMTS-4-D-Au	ADAMTS-4-specific peptide conjugated with fluorescent dye for enzyme detection.
	Inflammatory mediators response	TKCP@DEX	ROS-sensitive NPs target cartilage via thioketal-linked peptides.
	Inflammatory mediators response	pPADNs	PBA-induced pPAD forms melanin-like structures for PA imaging.
	Inflammatory mediators response	DAF-FM, NM-LANPs@Ru	NO monitoring showed by fluorescence.
	Inflammatory mediators response	DCNP@SeTT, NIR-ClO	Exhibiting rapid and specific responsiveness to HClO.

### Smart materials for contrast-enhanced computed tomography (CECT)

CECT garners considerable attention due to its ability to perform real-time, quantitative analyses of bone tissues using a widely accessible and cost-effective method.^32^ This capability is enhanced by the use of intelligent NPs. Currently, the contrast agents employed consist of small anionic molecules, which lack targeting specificity and are repelled by the negatively charged cartilage matrix.^33^ This interaction hinders their distribution within the tissue, resulting in suboptimal CT attenuation values.^34^ Therefore, there is a need for optimally charged cationic contrast agents that offer greater specificity.

Tantalum pentoxide (Ta_2_O_5_) NPs are promising materials for use as contrast agents in CT imaging due to their excellent X-ray radiation attenuation properties and biocompatibility in murine models.^35^ Freedman et al. synthesized Ta_2_O_5_ NPs functionalized with ammonium ions, enabling them to target the anionic glycosaminoglycans (GAGs) present in cartilage tissue.^36^ These NPs were utilized to assess distal metacarpophalangeal (MCP) cartilage defects in human cadaveric specimens, exhibiting preferential absorption in defect regions. When injected into rat knee joints, the NPs demonstrated effective cartilage imaging and in vivo safety. Additionally, Lawson et al. developed tantalum oxide (TaO) NPs encapsulated in trimethylammonium ligands. The CECT attenuation values obtained with these NPs were found to correlate with both GAG concentration and tissue stiffness.^32^

**Fig. 3:**
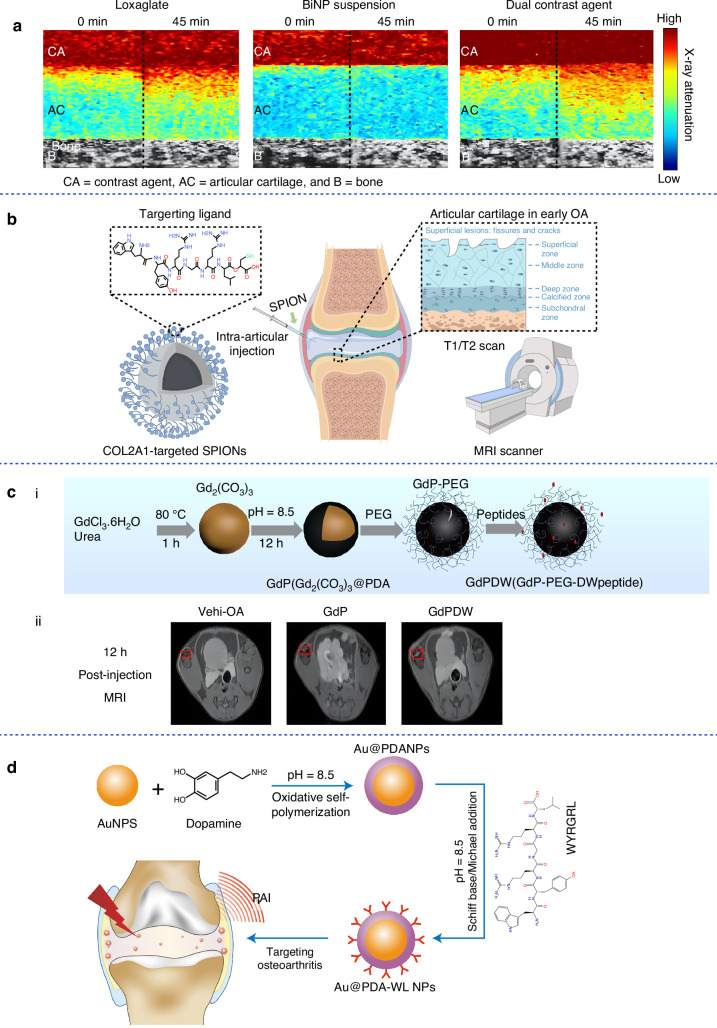
Materials designed for imaging-based diagnosis of OA. **a** CT images of an enzymatically degraded osteochondral sample using IOX, BiNP, and dual contrast agent at different time points. Reproduced with permission.^38^ Copyright 2017, Springer Nature. **b** Schematic illustration of COL2A1-targeted SPIONs for the enhanced MRI of articular cartilage. Reproduced under terms of the CC-BY license.^47^ Copyright 2023, The Authors, published by OXFORD UNIV PRESS. **c** (i) Schematic illustration of the prepared GdPDW NPs. (ii) T1-MR images of OA knee following intra-articular injection of the NPs. Reproduced with permission.^49^ Copyright 2019, Elsevier. **d** Schematic illustration of the synthesis of Au@PDA-WL NPs and their application in PAI. Reproduced with permission.^52^ Copyright 2024, Royal Society of Chemistry

The adoption of bismuth in CT contrast imaging is driven by its superior radiation attenuation properties, favorable toxicological profile, and economic viability.^37^ Saukko et al. engineered a hybrid contrast agent by conjugating ioxaglate (IOX) to bismuth oxide NPs (BINPs), achieving enhanced interfacial contrast for high-resolution cartilage segmentation in joint imaging (Fig. 3a).^38^

Additionally, non-metallic materials combined with contrast agents present an alternative approach. Zhang et al. developed agents by conjugating a cartilage-targeting cationic peptide carrier (CPC) and a multi-arm avidin nanostructure (mAv) to IOX. These agents demonstrated the capability to penetrate the entire cartilage thickness within 6 h through electrostatic interactions.^39^ Notably, these innovative agents produced CT imaging signals comparable to those of anionic IOX at ~40 times lower dosage.^39^

### Smart materials for MRI-based diagnosis of OA

The inflammation of the synovial membrane is a defining feature of OA.^40^ Catabolic and pro-inflammatory mediators, including cytokines, nitric oxide (NO), prostaglandin E2 (PGE2), and neuropeptides, are produced by the inflamed synovium, disrupting the equilibrium between cartilage matrix degradation and repair. This disruption results in the excessive production of proteolytic enzymes that contribute to cartilage breakdown. Furthermore, cartilage alterations exacerbate synovial inflammation, establishing a self-perpetuating cycle.^41^ Imaging of cartilage is crucial for the early diagnosis of OA, with MRI offering potential assistance in this regard.^42^ However, the lack of blood vessels in articular cartilage and the specific binding properties of cartilage present significant challenges for contrast agents to overcome.

Recent advancements in the development of intelligent NPs provide promising solutions to existing challenges by effectively identifying mediators. For example, targeted superparamagnetic iron oxide NPs (SPIONs) have been engineered as specialized contrast agents for precise targeting.^43^ These particles are designed to avoid infiltration into healthy cartilage while specifically accumulating in areas of structural cartilage damage, thereby highlighting regions of therapeutic interest. Contrast agents capable of targeting bone tissue present the potential for imaging bone metabolic activity using MRI. The incorporation of bisphosphonates (BP) onto SPIONs has attracted significant attention.^44^ To enhance bone affinity, Panahifar’s team conjugated alendronate (ALN)-a bisphosphonate with high specificity for calcium-rich hydroxyapatite-onto SPIONs, enabling targeted accumulation in mineralized bone matrices.^45^ These bone-targeted SPIONs had been proposed as non-ionizing contrast agents for MRI-based bone imaging, offering an alternative to traditional radioisotopes. In vitro studies demonstrated that ALN-functionalized SPIONs exhibited 65% selective binding affinity toward hydroxyapatite, enabling real-time visualization of bone remodeling dynamics-a critical capability for diagnosing and monitoring metabolic bone pathologies. Rothenfluh et al. employed phage display of a peptide library to identify suitable targeting ligands through biopanning on denuded cartilage.^46^ In the study, the ligand WYRGRL was selected and was shown to bind to collagen II α1. Wu et al. modified SPIONs with the peptide ligand WYRGRL (resulting in a particle size of 5.9 nm), which enabled them to bind specifically to type II collagen within the cartilage matrix and enhance probe retention (Fig. 3b).^47^ As type II collagen was progressively lost during the development of OA, the binding of these peptide-modified ultra-small SPIONs to type II collagen decreased in OA cartilage, resulting in distinct magnetic resonance signals compared to healthy tissue.

Gadolinium (III) is a lanthanide element that can enhance the T1-weighted MRI signal. In addition to WYRGRL, a chondrocyte-affinity peptide was identified, which could effectively interact with chondrocytes in a specific manner, without any species specificity.^48^ Ouyang et al. reported a new Gd_2_(CO_3_)_3_-based NPs. After coating polydopamine (PDA) onto the Gd_2_(CO_3_)_3_ core, they further anchored a chondrocyte-affinity peptide (GdPDW), which exhibited excellent cartilage affinity and MR imaging suitability (Fig. 3c).^49^ Bone-targeted NPs present a promising opportunity as non-ionizing MRI contrast agents, enabling the visualization of dynamic bone turnover. This capability can be utilized in the diagnosis and ongoing monitoring of metabolic bone diseases and associated bone pathologies.

Manganese (Mn)-based nanomaterials, characterized by their low toxicity, catalytic properties, and MRI imaging capabilities, have attracted considerable academic interest. In a study by Lin et al., carboxymethyl chitosan (CMC) was conjugated with a cartilage-targeting peptide to synthesize CMC-assisted MnOx NPs.^50^ These NPs demonstrated excellent biocompatibility and a T1 relaxivity of 1.72 mM^−1^·s^−1^. Prior to the administration of contrast agents, the articular cartilage was scarcely visible across all groups in a rat model of early OA induced by destabilization of the medial meniscus (DMM). However, 24 h post-injection of the NPs, a marked increase in signal intensity was observed at the sites of cartilage damage.

### Smart materials for photoacoustic imaging

PAI is increasingly employed in the diagnosis and treatment of diseases due to its advantages, including highly selective excitation, absence of radioactive damage, and deep tissue penetration.^51^ Numerous nanomaterials have been utilized as exogenous photoacoustic contrast agents to enhance the signal-to-noise ratio of PA imaging, with particular efficacy in selectively highlighting images of articular cartilage over other tissues.

Gold NPs (AuNPs) are considered the most effective inorganic biomaterials for PAI, owing to their large surface area, favorable biocompatibility, low toxicity, and high thermal conversion efficiency. A novel nanoprobe was developed by coating AuNPs with PDA (Au@PDA NPs) and subsequently conjugating the WYRGRL peptide (Au@PDA-WL NPs) onto its surface.^52^ These plasma nanoprobes were capable of targeting collagen II and facilitating the aggregation of AuNPs on the surface of the articular cartilage matrix. This aggregation significantly enhanced the absorption cross-section of the nanoprobes in the near-infrared (NIR) region, thereby demonstrating superior PAI capabilities (Fig. 3d).

OA is a significant contributor to chronic pain in the elderly population. In response to this, Au et al. developed a nanoprobe composed of gold nanorods coated with molybdenum disulfide nanosheets (MoS_2_-AuNR), specifically designed to target nerve growth factor (NGF), a crucial mediator in pain perception.^53^ The MoS_2_ coating notably enhanced the photoacoustic and photothermal properties of the gold nanorods. By conjugating these MoS_2_-AuNR nanoprobes with NGF antibodies, they were able to selectively target painful OA knees in mouse models. The functionalized nanoprobes demonstrated preferential accumulation in the OA-affected knee compared to the contralateral healthy knee, with accumulation levels directly proportional to the severity of mechanical allodynia. In contrast to exogenous PAI contrast agents, endogenous contrast agents are more desirable due to their biocompatibility and biodegradability in vivo.^54^ Xiao et al. introduced endogenous melanin NPs (MNPs) encapsulated with poly-L-lysine (PLL) as positively charged contrast agents for PAI of cartilage.^55^ In vitro PAI studies revealed that PLL-MNPs exhibited greater cartilage uptake and longer retention times than anionic MNPs, with a positive correlation to GAG content in cartilage.

### Intelligent materials for biomarker detection in OA diagnosis

Biochemical markers are critical indicators for elucidating the pathophysiology of OA and predicting structural alterations in the joint. Cartilage affected by inflammation is marked by the overexpression of inflammatory factors, including matrix MMP-13 and ADAMTS-4, among others. With the advance of detection means, the precise identification of inflammatory markers can facilitate the accurate diagnosis of OA.

**Fig. 4:**
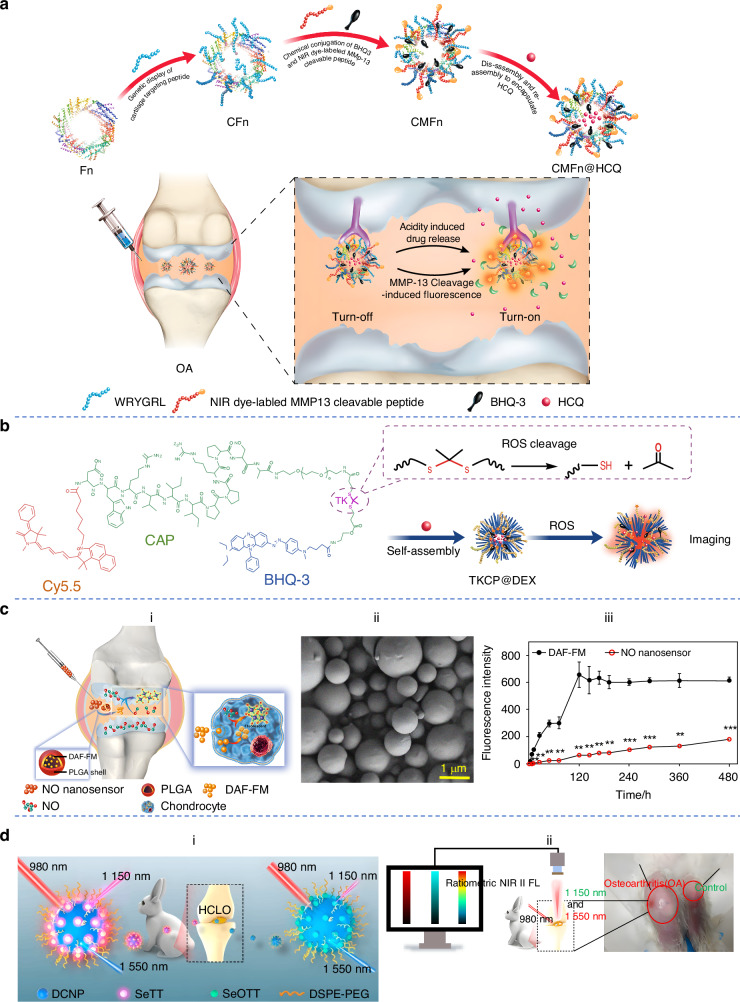
Materials designed for sensing biomarkers of OA. **a** Schematic illustration of CMFn@HCQ as a cartilage-targeting and MMP-13/pH dual-stimuli activatable theranostic nanoprobe for MMP-13 imaging of OA. Reproduced with permission.^56^ Copyright 2019, Elsevier. **b** Schematic illustration of synthesis of the self-assembly of ROS-responsive NPs. Reproduced under terms of the CC-BY license.^61^ Copyright 2021, The Authors, published by BioMed Central Ltd. **c** (a) Schematic illustration of NO nanosensors for predicting OA development; (b) Scanning electron microscope imaging of nanosensors; (c) Stability of NO nanosensors versus DAF-FM probe. Reproduced with permission.^63^ Copyright 2017, American Chemical Society. **d** (a) Schematic illustration of HCLO-responsive nanoprobe DCNP@SeTT@PEG; (b) Imaging of OA of rabbit using the DCNP@SeTT@PEG; Reproduced with permission.^65^ Copyright 2020, American Chemical Society

A pioneering design has emerged with biocompatible, cartilage-targeting, and dual MMP-13/pH-sensitive ferritin nanocages (CMFn).^56^ These NPs were modified with the peptide WRYGRL, thus generating cartilage-targeted ferritin nanocomposites. Then they conjugated MMP-13 cleavable peptide substrates with imaging dyes to form MMP-13-sensitive ferritin nanoprobes. The detected light signal intensity correlated with the severity of OA (Fig. 4a). Poly (2-ethyl-2-oxazoline)-poly (ε-caprolactone) (PEOz-PCL or PPL) is often used to develop pH-responsive drug carriers. Lan et al. modified PPL with type II collagen-targeted peptide to produce a cartilage-targeted polymer (C-PPL).^57^ PPL was conjugated with a peptide substrate for MMP-13 and a fluorescent dye (MR-PPL). Multifunctional nanomicelles sensitive to MMP-13/pH were obtained through self-assembly of C-PPL and MR-PPL. In the OA environment, MMP-13 enzymes cleaved their substrate on the micelles, and then enabled a strong fluorescence signal, thereby indicating the OA condition. Zhou et al. prepared MMP-13 enzyme-activated, cRGDfK-modified micelles and found it could diagnose the early-stage OA induced by DMM surgery on the knees.^58^

ADAMTS-4 plays a critical role in degrading aggrecan, an early event in the progression of OA. Peng et al. innovated a fluorescent probe grounded on AuNPs conjugated with a peptide specifically designed to detect the ADAMTS-4 enzyme.^59^ A nearly 3-fold increase in fluorescent intensity was observed in response to ADAMTS-4 at pM level. Additionally, ADAMTS-4 activity in the synovial fluid from 11 knee surgery patients was detected by the ADAMTS-4-D-Au probe. Compared to the chronic joint injury and end-stage OA groups, the fluorescent intensity in the acute joint injury group increased significantly, suggesting that this system may perform as a novel detection method for ADAMTS-4 activity. Using the rabbit model of early-stage OA and samples from OA patients, Liu et al. evaluated the diagnostic utility of AuNP probe for detecting mild chondral damage.^60^ Using both the AuNP probe and MRI together would provide better diagnostic accuracy than using MRI alone, thus improving the detection of mild cartilage damage.

In addition to the inflammatory factors, other products can also be the recognized target of sensing NPs. For example, inflammatory responses are typically associated with excessive production of ROS. Based on the association between the overproduction of ROS and the progression of OA, Shen et al. selected ROS as a biomarker and designed a cartilage-targeted and ROS-responsive nanoprobe (TKCP@DEX).^61^ The fluorescence signal was strong in the OA microenvironment but very weak in normal chondrocytes and joints, indicating that the level of the fluorescence signal was related to the content of ROS (Fig. 4b). Zhao et al. synthesized a PEG modified, phenylboronic acid (PBA) functionalized L-DOPA pro-antioxidant (pPAD), which could self-assemble into NPs (pPADNs).^62^ PBA was an efficient ROS-reactive group that allowed pPAD to convert into active L-DOPA upon oxidant trigger and further form melanin-like structures, which possessed PA imaging properties. The knee joints of healthy rats showed consistently low PA signals at all studied time points. However, the knee joints of the OA model group displayed an initial low PA signal, with the intensity increasing over time. Jin et al. documented the development of a NO nanosensor rooted in poly(lactide-co-glycolic acid) (PLGA) NPs, aimed at identifying the over-production of this inflammatory mediator (Fig. 4c).^63^ Upon encountering NO in the environment, NO-sensing molecules underwent a transformation into a fluorescent state, enabling visualization through fluorescence microscopy. In vitro, the nanosensor enabled the monitoring of NO release in interleukin-1β-stimulated chondrocytes. In the rat model of OA, it allowed for the quantification of NO levels in joint fluid. Besides, an aggregation-induced emission (AIE) probe, which was responsive to NO, could also smartly monitor the progression of OA both in vitro and in vivo.^64^ A novel NIR-II fluorescence molecule, 4,7-bis (5-(4-(diphenylamine)phenyl)-2-thiophene) [1,2,5]-selenadiazolo [3,4f] benzo [c][1,2,5] thiadiazole (SeTT), was reported to exhibit rapid activation and high specificity toward hypochlorous acid (HClO).^65^ To obtain a NIR-II ratiometric nanoprobe, SeTT was encapsulated on the surface of down-conversion nanoparticles (DCNP), achieving the nanoprobe DCNP@SeTT. The detection limitation was 0.4 μmol/L. DCNP@SeTT was used for the monitoring of HClO in vivo in the rabbit model of OA. And it exhibited clear fluorescence imaging signals through thick tissue in the big animal model (Fig. 4d). A smart HClO-responsive probe, DHU-CBA1, was rapidly activated by HClO within an extremely short time of 80 s, demonstrating good selectivity and sensitivity.^66^ Luo et al. developed a new mitochondria-targetable NIR fluorescent probe, NIR-ClO, for the specific analysis and imaging of HClO levels associated with the OA.^67^ In the presence of HClO, NIR-ClO achieved high sensitivity and selectivity due to the HClO-triggered specific C=C bond cleavage reaction, with a low detection limit (LOD) of 28.3 nmol/L and rapid response time (<60 s), making it suitable for HClO detection. Furthermore, NIR-ClO had been successfully used for imaging HClO in live RAW264.7 cells and in a rat model of OA.

### AI in OA diagnosis

The interpretation of diverse medical imaging modalities, such as PET, CT, and MRI, is a labor-intensive task that heavily relies on the expertise of physicians. However, AI is progressively revolutionizing the field of pathological diagnosis. AI applications, particularly those involving advanced medical image processing, enhance the accuracy and efficiency of image recognition and classification, thereby significantly improving the precision and efficacy of diagnostic procedures.^68^

Nonetheless, the foundational rationale for diagnosis remains reliant on image characteristics. The detection of subtle pathological features, particularly in the early stages, remains beyond the current capabilities of AI. Encouragingly, the emergence of advanced materials holds the potential to enhance the sophistication of AI-driven diagnostic processes. Plasmonic biosensors have garnered significant interest in the cutting-edge field of exosome detection, attributed to their label-free, real-time, and high-sensitivity attributes. The integration of plasmonic biosensors with exosomes presents substantial advantages in the analysis of exosome-based spectral and image signals for disease diagnostics. The application of representative machine learning (ML) algorithms in plasmonic biosensing research for exosome liquid biopsy enables the efficient processing of complex exosome datasets and the development of robust predictive models for accurate diagnosis.^69^ Additionally, certain nanoengineered three-dimensional sensors, equipped with ultra-small nanoscale probes, can be trained using artificial neural networks (ANNs) to accurately identify lesion locations.^70^ While these technologies are still emerging in the field of OA diagnosis, intelligent materials are enhancing the functionality and accuracy of AI-based diagnostic methods. Rong et al. developed a superparamagnetic composite hydrogel scaffold incorporating FeO NPs, which enables the monitoring of osteochondral defect repair via MRI.^71^ Moreover, the incorporation of emerging technologies has markedly propelled the advancement of AI-driven diagnostic tools. By harnessing progress in microfluidic systems and organ-on-a-chip technologies, in vitro detection of inflammatory cells has been successfully accomplished.^72^ A three-dimensional synovium-on-a-chip model was developed to investigate the initiation and progression of inflammatory responses within synovial tissue. This study utilized light scattering techniques to noninvasively evaluate dynamic responses at the synovial tissue level upon exposure to proinflammatory cytokines. These responses encompassed the remodeling of synovial network architecture and alterations in organoid condensation, both of which were modulated by cadherin-11-mediated cell-cell adhesion.^73^ This innovation has the potential to assist AI in identifying key areas of interest, thereby reducing MRI acquisition time through the use of accelerated MRI and super-resolution imaging techniques.^74^

## Smart materials for OA therapy

The application of biomaterials in OA treatment has undergone significant evolution, characterized by increasing structural complexity and sophistication. Initially, biomaterials were regarded primarily as passive carriers for pharmaceutical agents. However, recent advancements in research have endowed these materials with a variety of enhanced properties, enabling them to function as targeted, activatable, and controllable systems, ensuring that drug delivery is safe, flexible, accurate and more controllable. These advancements allow for more intelligent modulation of the therapeutic process through external stimuli or internal variations. A recent review systematically elaborated on the innovative approaches in smart drug delivery systems and smart scaffolds for the repair and regeneration of articular cartilage, providing a comprehensive categorization of smart biomaterials utilized in cartilage repair and regeneration.^23^

**Fig. 5:**
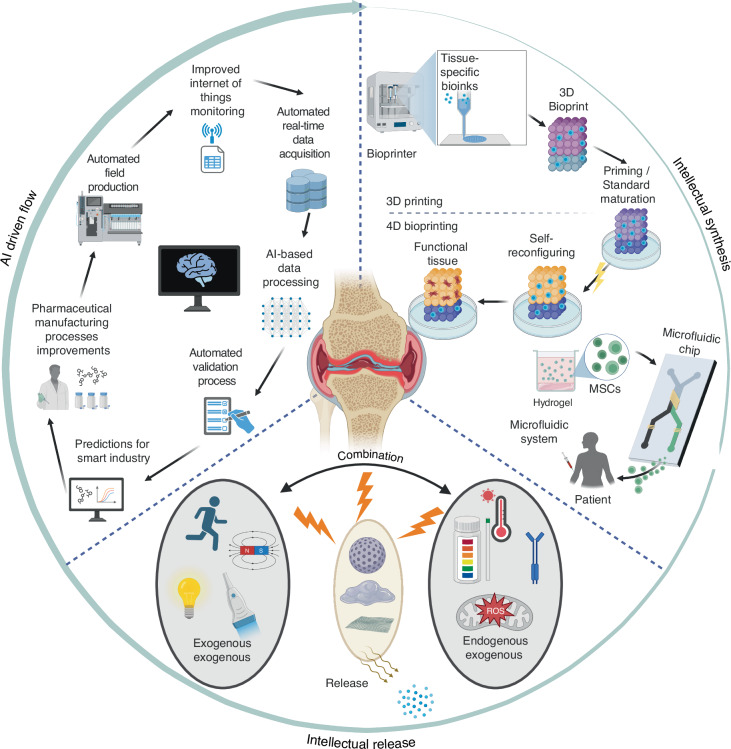
Schematic illustration of the evolution of biomaterials for OA therapy. Created with BioRender.com

However, the repair of cartilage is just a small part of OA treatment, the development of intelligent material runs through the entire therapy. The evolution of diverse micro-structured materials signifies a substantial shift from the use of singular materials, such as NPs and hydrogels, to the development of smart materials and stimuli-responsive delivery systems. Furthermore, AI technology has been progressively employed to accomplish the initial objective of designing single-functional biomaterials and has subsequently been utilized to address complex multi-objective challenges. This progression marks a pivotal advancement in the integration of intelligence from therapeutic processes to material design (Fig. 5). Our review examines this developmental trajectory comprehensively.

### Intellectualization of the release system of materials for OA treatment

Stimuli-responsive ability is the basic characteristic of smart materials. This material systems require the use of biocompatible materials that are sensitive to specific external (such as light, magnetism, ultrasound, and mechanical force) or internal (such as reactive oxygen species, pH, temperature, and enzymes) triggers to facilitate the intelligent release of drugs. The drug release process can be initiated through various disintegration mechanisms of the carrier material, including hydrolytic cleavage, specific protonation, and changes in molecular or supramolecular conformation.^23,75^ The therapeutic impact of the system, either initiated or enhanced by stimulation, possesses the benefits of both controllability and precision.

#### Exogenous stimuli

**Fig. 6:**
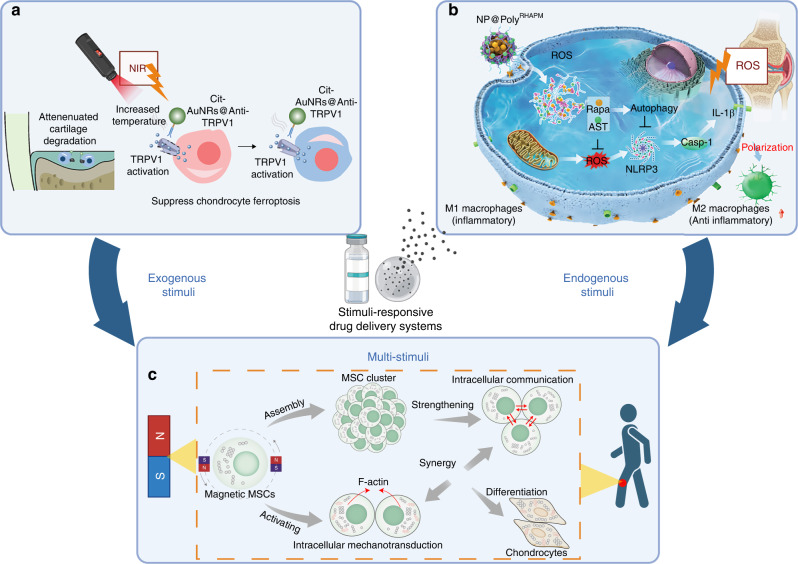
Biocompatible materials that are responsive to stimulation. **a** Illustration of Cit-AuNRs@Anti-TRPV1 switch for photothermal activation of TRPV1 signaling to attenuate OA. Reproduced under terms of the CC-BY license.^76^ Copyright 2024, The Authors, published by Wiley-VCH Verlag. **b** Schematic illustration of NP@Poly (RHAPM) influencing macrophage polarization in knee OA in response to ROS. Reproduced with permission. Reproduced under terms of the CC-BY license.^104^ Copyright 2024, The Authors, published by Wiley-VCH Verlag. **c** Diagram illustrating magneto-mechanical force-induced chondrogenic differentiation in Magcells. Reproduced with permission.^112^ Copyright 2023, American Chemical Society. Created with BioRender.com

Light, encompassing the ultraviolet (UV), visible, and NIR spectrums, represents a promising exogenous stimulus for regulating therapeutic processes due to its precise controllability, which is achieved by meticulously adjusting beam parameters. Considering the thermally activated nature of TRPV1, citrate-stabilized gold nanorods (Cit-AuNRs) are conjugated to a TRPV1 monoclonal antibody (Cit-AuNRs@Anti-TRPV1) to function as a photothermal switch for TRPV1 activation in chondrocytes under NIR irradiation. This approach attenuates cartilage degradation by suppressing ferroptosis in chondrocytes (Fig. 6a).^76^ Photothermal therapy plays a crucial role in the implementation of advanced drug release mechanisms for the regeneration of articular cartilage. In a significant study, Xue and colleagues developed a dual-drug delivery system based on a metal-organic framework (MOF)-decorated mesoporous polydopamine (MPDA) structure. The system consisted of rapamycin (Rap) encapsulated within the mesopores and bilirubin (Br) adsorbed onto the shell of the MOF. These delivery agents were capable of sequential release upon NIR laser stimulation.^77^ Additionally, the remarkable photothermal properties of the phase-change material enabled the NIR-triggered release of kartogenin (KGN), thereby enhancing cartilage repair.^78^

Mechanical forces, such as compressive, tensile, and shear forces, which were traditionally considered as promoters of mechanically sensitive proteins, can be harnessed as signals for stimulus response in drug release or signal transmission processes.^79,80^ Mechanoresponsive drug release systems have the capability to respond and adapt to the dynamic mechanical microenvironment of articulating joints.^81^ For example, the design of mechanically-activated microcapsules is intended to facilitate the targeted delivery of therapeutics in response to the mechanical loading conditions characteristic of regenerating musculoskeletal tissues, thereby enhancing tissue repair processes. The physical properties of these microcapsules can be modulated to regulate the release of therapeutic agents within maturing repair tissues subjected to dynamic loading within the joint.^82^

Magnetism is regarded as an optimal exogenous stimulus due to its minimal interaction with biological tissues while ensuring effective penetration.^83,84^ Functionalized magnetic NPs hold promise as efficient carriers in advanced drug delivery systems, which can be controlled by either a static or dynamic magnetic field.^75^ Moreover, the γ-Fe_2_O_3_@TA@ALN MNPs, comprising γ- Fe_2_O_3_, tannic acid (TA), and ALN, are designed to reconstruct the osteoarthritic microenvironment and attenuate the progression of TMJOA. When exposed to a 0.2 T static magnetic field, these γ- Fe_2_O_3_@TA@ALN MNPs enhance the synthesis of cartilage-specific proteins while inhibiting the expression of catabolic-related genes and the generation of reactive oxygen species, thereby providing additional protection to the temporomandibular joint cartilage.^85^ In the study undertaken, researchers developed magnetic polysaccharide hydrogel particles to serve as microcarriers for the synergistic treatment of OA. When subjected to a magnetic field, the microcarriers released dexamethasone (DS) and exosomes (Exo), which collectively exhibited a synergistic effect in mitigating OA symptoms and facilitating cartilage repair.^86^

Ultrasound serves as an external mechanical force stimulus in advanced drug delivery systems aimed at articular cartilage repair, due to its high degree of controllability, non-invasive characteristics, and superior tissue penetration capabilities.^84^ Initially, therapeutic agents are encapsulated within a polymer capsule that is sensitive to ultrasound. These encapsulated agents are then targeted to the articular cartilage surface through the application of ultrasound waves. Ultimately, the thermal and/or mechanical effects induced by radiation forces or cavitation phenomena enhance the release of therapeutic agents.^75,84^ Jahanbekam was a pioneer in developing an injectable thermo-ultrasound-triggered drug delivery system, employing Pluronic® F-127, hyaluronic acid (HA), and gelatin. This novel carrier system not only has the potential to prolong the duration of drug release but also offers the capability to adjust the rate of drug release in response to ultrasound stimulatio.^87^ Furthermore, PLGA microparticles (MPs) encapsulating KGN, referred to as KGN@MPs, were developed as an ultrasound-responsive drug delivery system for applications in articular cartilage tissue engineering. Upon exposure to ultrasound waves, the PLGA MPs exhibited a significant structural collapse, facilitating the gradual release of KGN from the MPs.^88^

#### Endogenous stimuli

The endogenous stimulus functions as a frequently employed catalyst for the release or activation of biomaterials. Variations in joint pH, induced by an inflammatory response, can be leveraged as a mechanism for drug delivery.^75^

The pH level within the joint is reduced to ~6.0 as a result of the inflammatory response, and it may occasionally decrease to below 5.0. Consequently, this reduction in pH within damaged joints can be exploited as a mechanism to trigger drug delivery.^89,90^ In the design proposed by Tajik, glucosamine was converted into nanoglucosamine via the ionic gelation method, utilizing sodium tripolyphosphate (TPP) as a cross-linking agent to improve enzymatic stability. The resulting NPs exhibited sustained drug release characteristics, which were contingent upon pH levels.^91^ Furthermore, Wang et al. developed an innovative hierarchical drug delivery system with pH-responsive characteristics to ensure thorough penetration of the cartilage extracellular matrix, thereby enhancing therapeutic efficacy.^92^ In addition, a pH-responsive biodegradable hollow-structured manganese Prussian blue nanozyme was also engineered for the treatment of osteoarthritis.^93^

In addition to pH-responsive systems, enzymes are integral to the development of biomaterials. The enzyme-responsive nano-drug delivery system comprises two primary components: (i) a nanomaterial scaffold incorporating an enzyme-sensitive segment that disassembles upon enzyme interaction, and (ii) encapsulated therapeutic agents that are either linked to the carrier by biodegradable bonds, such as ester bonds, or associated through electrostatic interactions between the active ingredient and the charged carrier, or bound to an external lipid membrane or lipid core.^94^ For instance, Joshi et al. documented the creation of a hydrogel platform capable of controlled disassembly and drug release, dependent on the enzyme concentrations expressed during arthritis exacerbations.^95^ The TG-18 hydrogel demonstrates the ability to disassemble in the presence of flare-associated enzymes, notably MMPs, which are abundant in inflamed joints. Additionally, peptides sensitive to MMP-13 may act as catalysts for the degradation of microspheres, thus regulating the release of anti-inflammatory agents, such as hydroxychloroquine, within the OA tissue microenvironment.^96^

The inflammatory response and joint movement can cause a modest elevation in the temperature of the joint cavity (~2.0–3.1 °C), serving as an endogenous trigger for controlled drug release.^97^ Thermoresponsive biomaterials, characterized by a lower critical solution temperature (LCST), typically undergo structural transitions in response to temperature fluctuations. These materials often comprise liposomes, polymer micelles, or nanoparticles.^75^ The amphiphilic properties and the ability of these materials to degrade into fully soluble compounds make thermoresponsive polymers particularly attractive for applications in drug delivery and tissue engineering.^98^ Recent studies have demonstrated the development of thermo-responsive polymeric nanospheres that facilitate simultaneous and independent dual drug delivery in response to temperature changes. These nanospheres enable the immediate release of diclofenac alongside the sustained release of KGN, with both release profiles being independently regulated by temperature fluctuations.^99^ Guan et al. developed polymeric micelles or NPs utilizing poly(N-isopropylacrylamide) (PNIPAM) hydrogel, which exhibit the capacity to swell or shrink in response to a physiologically relevant LCST ranging from 31 to 33 °C. This property enables the modulation of drug release through thermal responsiveness. For instance, PNIPAM microgels were functionalized with negatively charged poly(3-sulfopropyl methacrylate potassium salt) (PSPMK) brushes, integrating joint lubrication with thermo-responsive drug release to facilitate the repair of articular cartilage.^100^ Nevertheless, PNIPAM is increasingly being supplanted by alternative thermo-responsive polymeric materials owing to its limited biodegradability. Recent studies have reported the development of thermoresponsive nanospheres (F127/COS/KGNDCF), which consist of an outer crosslinked polyethylene oxide chain, dicarboxylate-conjugated chitosan oligosaccharide (COS), and pluronic F127, along with an inner KGN-conjugated polypropylene oxide network.^99^

The incidence of arthritis elevates the levels of ROS within cells and their microenvironment, thereby altering redox conditions, which can subsequently trigger drug release.^101^ Based on the underlying pathological mechanisms, redox-responsive nanomaterials experience degradation, structural modifications, functional regulation, or alterations in their physical and chemical properties. These processes facilitate the targeted delivery and release of therapeutic agents at the lesion site.^102^ According to recent studies, the fracture mechanism of functional groups in redox-responsive materials is activated by redox substances, such as ROS or glutathione (GSH).^103^ Consequently, ROS and GSH serve as significant indicators of inflammation and are utilized to initiate drug release, thus enabling intelligent control of therapeutic systems.^104^ Upon activation by ROS, the NPs restored mitochondrial membrane potential, increased GSH levels, and stimulated intracellular autophagy, effectively repolarizing M1 macrophages to the M2 phenotype (Fig. 6b).

#### Multi-stimuli-responsive drug release

The implementation of an advanced drug release system can significantly enhance the efficiency of drug delivery and reduce the adverse effects on joint tissues.^75^ Nevertheless, OA represents a complex pathogenetic process, often associated with a range of changes including variations in pH, ROS, and MMPs.^84^ A single-stimulus approach is insufficient for the precise release of medication in lesions characterized by complex microenvironments and multiple signaling pathways.^105^ Enhancing sensitivity to multiple stimuli can increase the specificity and versatility of triggered delivery systems for drugs and bioactive factors.^106^ This multi-stimulation can be the multiple endogenous stimuli or the combination of internal- external stimulus which make the biomaterial more intelligent.

Endogenous stimuli can be harnessed to develop multi-stimuli-responsive drug delivery systems, thereby enhancing the sensitivity to environmental conditions. Piezoelectric biomaterials exemplify force-activated materials that demonstrate biocompatibility, biodegradability, and durability.^107^ Notably, a biodegradable piezoelectric poly(L-lactic acid) (PLLA) nanofiber scaffold has been designed to act as a self-sustaining electrical stimulator, promoting chondrogenesis and cartilage regeneration in response to mechanical force or joint loading.^108^ Moreover, the integration of two endogenous stimuli-responsive systems holds the potential to improve therapeutic outcomes significantly. This potential was demonstrated by researchers who developed a nano-micelle-based stimuli-responsive nanoplatform, wherein drug release was precisely regulated by acidic pH and MMP-13, specifically targeting OA therapy.^56,57^ Given the pathological alterations in pH and ROS within the OA microenvironment, the dual-responsive strategy targeting pH and ROS has garnered significant attention in OA treatment. When integrated with hydrogels, these smart materials can adapt to the complex joint environment, thereby facilitating multiple functions such as extracellular RNA scavenging, drug release, and microenvironment remodeling, all of which contribute to accelerated healing processes.^109,110^

In addition to dual endogenous stimulation, the combination of endogenous and exogenous stimuli often enhances the controllability of drug release. An injectable, biodegradable piezoelectric hydrogel composed of short electrospun poly-L-lactic acid nanofibers embedded within a collagen matrix has been developed. This hydrogel can be administered into joint spaces and is capable of generating localized electrical stimuli upon ultrasound activation, thereby facilitating cartilage repair. Empirical evidence indicates that the application of ultrasound to the piezoelectric hydrogel significantly enhances cell migration and stimulates stem cells to secrete transforming growth factor-beta 1 (TGF-β1), which is instrumental in promoting chondrogenesis.^111^ Moreover, the integration of intercellular mechanical communication and mechanosignaling processes may enhance cell-cell interactions, thereby providing a foundation for the development of mechano-therapeutics targeting cartilage repair in OA treatment (Fig. 6c).^112^ In this study, researchers developed magnetic polysaccharide hydrogel particles to serve as microcarriers for the synergistic therapy of OA. When subjected to magnetic fields, the release of DS and Exo from these microcarriers exhibited a synergistic effect in mitigating OA symptoms and facilitating cartilage repair.^86^

### Advanced synthesis of materials for OA treatment

The traditional methods for synthesizing and assembling materials typically rely on manipulating physical or chemical processes, such as simple casting or droplet drying techniques.^113^ However, as the demand for precision in biomaterials increases, controlling primary processing parameters-such as suspension rheology, surface roughness, evaporation rate, and contact angle hysteresis of the droplet-substrate interface-may not satisfy the necessary requirements.^114^ Consequently, there is an urgent need for the intellectualization of these processes to achieve improved therapeutic outcomes in complex microenvironments, such as joint spaces.

Over the years, researchers have explored various innovative approaches to enhance production efficiency, including 3D printing, microfluidics, and 4D printing. These advanced biomaterials consistently exhibit distinct characteristics compared to their traditional counterparts. The evolution in the synthesis of biomaterials signifies the advancement in the smart materials, fulfilling the requirement of precision, controllable and self-regulating.

#### 3D printing

3D printing technologies, also referred to as additive manufacturing (AM) or 3D rapid prototyping technologies-including binder jetting, directed energy deposition, material extrusion, material jetting, powder bed fusion, sheet lamination, and vat photopolymerization-constitute innovative and emerging methodologies for replicating the essential structural and functional characteristics of various human tissues using patient-specific medical imaging. It has long been used in bone tissue engineering.^115^ However, these technologies also offer a means to surpass the limitations inherent in traditional 3D scaffold manufacturing techniques such as electrospinning, freeze-drying, gas foaming, and particle/porogen leaching.^116,117^ It demonstrates benefits in minimizing waste, eliminating the need for time-intensive tooling engineering, and facilitating geometrical, structural, and compositional design processes.^114^

Hydrogels have traditionally been considered optimal carriers for drug delivery. The rapid progress in nanotechnology and materials science has intensified efforts to develop safer, more personalized, and more efficacious hydrogels for addressing common yet complex inflammatory conditions.^118^ The integration of 3D printing technology has enhanced the performance of hydrogels in sustained release applications, thereby demonstrating greater potential in anti-inflammatory treatments. For instance, Ding et al. developed a robust hydrogel with a biomimetic microstructure, utilizing an emulsion-type photosensitive resin. This water-oil biphasic hydrogel ink facilitates the incorporation of both water- and lipid-soluble drugs, resulting in hydrogel scaffolds that exhibit sustained dual-drug release capabilities and possess superior mechanical properties with minimal swelling, owing to advancements in 3D printing technology.^119,120^ Furthermore, Zhang et al. successfully fabricated an antioxidative scaffold. The bioprinted scaffold exhibited a capacity to mitigate intracellular oxidative stress in embedded chondrocytes when exposed to H_2_O_2_, facilitated by secondary ionic crosslinking between the introduced calcium ions and the carboxylate groups within the alginate backbone.^121^ Additionally, other forms of scaffolds produced through 3D printing have demonstrated significant potential in the treatment of OA. Notably, silk-gelatin scaffolds exhibit superior efficacy in inhibiting OA progression.^122^

**Fig. 7:**
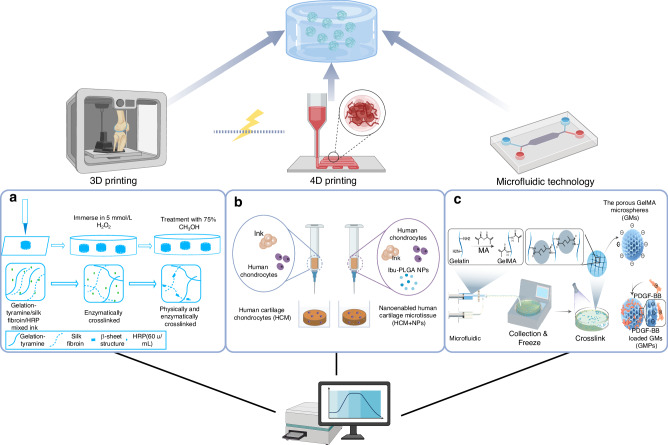
Intelligent synthetic technologies used in micro materials fabrication. **a** Schematic diagrams of the 3D SF-GT hydrogel scaffold synthesis. Reproduced under terms of the CC-BY license.^128^ Copyright 2021, The Authors, published by KeAi Communications Co. **b** The comparation of bioinks used in synthesizing nanoen-abled human cartilage microtissue and human cartilage microtissue. Reproduced with permission.^143^ Copyright 2024, John Wiley and Sons. **c** GMPs engineering through microfluidic synthesis of GMs followed by electrostatic loading of PDGF-BB. Reproduced with permission.^154^ Copyright 2023, John Wiley and Sons. Created with BioRender.com

Articular cartilage is a distinctive structure that facilitates low-friction articulation and aids in load transmission through the osteochondral unit, enabling it to endure high cyclic loads without undergoing degenerative changes.^123^ Regrettably, osteochondral degradation occurs progressively in the stages of OA.^124^ Consequently, the regeneration and repair of articular cartilage tissue are crucial in the treatment of OA. Scaffolds, serving as supportive structures, can repopulate damaged areas of articular cartilage and temporarily assume the role of the extracellular matrix (ECM) in the developing tissue.^125^ Furthermore, scaffolds serve as carriers for cells and delivery vehicles for drugs and bioactive factors, thereby mediating cellular activities that promote extracellular matrix deposition and the regeneration of articular cartilage tissue.^126^ The intelligent customization of scaffolds to address osteochondral defects has also garnered significant attention. Chen et al. engineered three-dimensional bioprinted xanthan gum (XG) hydrogels by functionalizing XG with methacrylic (MA) groups, aimed at applications in cartilage repair therapy. This innovative design not only demonstrates significant ROS scavenging capabilities, thereby safeguarding stem cells from oxidative stress, but also improves the mechanical properties of the resultant scaffolds.^127^ Moreover, Li et al. engineered a macroporous silk fibroin-gelatin (SF-GT) hydrogel scaffold through the horseradish peroxidase (HRP)-mediated crosslinking of silk fibroin (SF) and tyramine-substituted gelatin (GT), employing extrusion-based low-temperature 3D printing techniques. The macroporous architecture of the scaffolds significantly enhanced stem cell attachment and differentiation (Fig. 7a).^128^ Additionally, 3D printing offers the potential for multilayer treatment applications. Lee et al. developed two types of composite spheroids composed of human adipose-derived stem cells (hADSCs) and nanofibers functionalized with transforming growth factor-beta 3 (TGF-β3) and bone morphogenetic protein-2 (BMP-2) to induce chondrogenesis and osteogenesis, respectively. Subsequently, each type of spheroid was cultured within a 3D printed microchamber. This approach promoted the targeted differentiation of the transplanted stem cells in a spatially organized manner, thereby facilitating the formation of a bilayer structure characteristic of osteochondral tissue.^129^

The 3D-bioprinting technique employed in cartilage tissue engineering typically comprises three critical components: cells, growth factors, and printed scaffolds, which are constructed from diverse bioinks. The reparative mechanisms of these bioinks primarily operate through two pathways: (i) the printed constructs provide a provisional ECM environment that facilitates chondrogenesis and angiogenesis, thereby promoting the formation of new cartilage tissue; (ii) the engineered biomaterials substitute the damaged or absent cartilage, thereby restoring the functionality of the impaired joint.^130^ Inks derived from natural resources typically exhibit excellent biocompatibility; however, they often lack mechanical strength. Conversely, bioinks composed of synthesized polymers generally possess robust mechanical properties but may not be as biocompatible. The selection of appropriate bioinks is crucial to ensuring that printed constructs provide adequate mechanical and structural support as well as sufficient nutrient supply.^131^ Optimizing biomaterial inks also expands the potential for therapeutic applications. To produce functional and high-quality neocartilage, native progenitor cells and stem cells are frequently employed alongside cartilage scaffolds to enhance the repair of cartilage defects.^132^ For instance, Liu et al. developed a bone marrow-derived mesenchymal stem cell (BMSC)-laden 3D-bioprinted multilayer scaffold composed of methacrylated hyaluronic acid (MeHA) and polycaprolactone, incorporating KGN and β-TCP, for the repair of osteochondral defects in each region. This design not only facilitates chondrogenesis but also significantly enhances joint function.^133^ In addition to stem cells, cartilage powder and adipose tissue also possess the potential for cartilage repair. Jina et al. innovatively utilized a biomatrix composed of human costal-derived cartilage powder, micronized adipose tissue and fibrin glue. The 3D printing material demonstrated a substantial impact on reducing inflammation levels and promoting cartilage-like growth in the experimental group.^134^ Furthermore, the integration of immune-regulatory exosomes with 3D bioprinting hydrogel (3D-BPH-Exos) presents a promising approach for the immunomodulation of cartilage tissue and the treatment of OA. It has the potential to attenuate the formation of intracellular inflammasomes and the secretion of pro-inflammatory cytokines such as IL-1β, TNF-α, and INF-γ. Additionally, it may inhibit chondrocyte apoptosis by restoring mitochondrial function and promoting chondrogenesis in synovial mesenchymal stem cells, osteoprogenitor cells, and osteoclasts.

#### 4D printing

4D printing represents a sophisticated advancement in additive manufacturing technology, enabling the creation of constructs that can experience pre-programmed alterations in shape, properties, or functionality upon exposure to specific stimuli.^135^ This technology extends beyond traditional 3D printing by incorporating the fourth dimension of time. By introducing a temporal element, 4D printing facilitates the production of dynamic structures capable of responding to external stimuli, thereby transforming the static nature of conventional 3D printed objects.^136^ To date, 4D printed materials have been employed in the field of biomedical engineering for the design and fabrication of various biomedical devices, including stents, occluders, microneedles, smart 3D-cell engineered microenvironments, drug delivery systems, wound closures, and implantable medical devices.^137^ The application of 4D printing has the potential to enhance the intelligence of drug delivery systems. The fundamental aspect of 4D printing lies in the judicious selection of stimuli-responsive materials, such as shape memory polymers (SMPs), hydrogels, and liquid crystal polymers (LCPs), among others.^138^ The effective integration of these materials relies on a meticulously designed interaction mechanism, a suitable 3D printing technique, the accurate application of external stimuli, and biocompatibility. 3D printing technologies serve as the basis for 4D printing processes, including fused deposition modeling (FDM), direct ink writing (DIW), and direct laser writing (DLW), among others.^139–141^

With the increasing interest in personalized medicine, 4D printing has garnered significant attention for its potential to revolutionize advanced drug delivery systems, such as microneedles (MNs), implants, and scaffolds.^142^ Although it remains a relatively novel area within the context of drug delivery systems for OA therapy, this innovative and promising approach is beginning to gain traction in the chemical pharmaceutical industry. Couto et al. have utilized 4D-bioprinting to fabricate human cartilage microtissues (HCM) enhanced with ibuprofen-loaded poly(lactic-co-glycolic acid) NPs (ibu-PLGA NPs) to specifically target and mitigate localized inflammation and inhibit nerve sprouting. The nano-enabled HCM did not provoke a systemic immune response and effectively reduced the local recruitment of mature dendritic cells, as well as the secretion of various inflammatory mediators and matrix metalloproteinases (Fig. 7b).^143^

#### Microfluidic technology

Microfluidic technology, which involves the manipulation of distinct fluidic properties at the micrometer scale, offers promising solutions to the challenges associated with OA theranostics.^27,144^ As a versatile and robust platform, it has been employed across a diverse array of fields, including disease modeling and drug screening,^145^ diagnostics,^146^ biofabrication,^147,148^ and OA therapy.^149^ In the field of drug delivery, microfluidic technology enhances the processing of biomaterials, such as hydrogels and microgels, by providing a more efficient and sophisticated approach compared to traditional methods, which often suffer from uncontrollable characteristics such as variability in size, shape, and complex experimental setups. Broadly, the mechanisms for microgel formation within microfluidics can be divided into two categories, i.e. passive and active methods.^150^ Although active methods are predominantly employed for liquid manipulation, passive droplet formation is the primary microfluidic technique utilized for generating microgels. In the passive approach, droplets are generated through the emulsification of two immiscible fluids-ispersed and continuous-at the microfluidic junction. Three prevalent junction designs include co-flow, cross-flow, and flow-focusing configurations. In comparison to traditional methods, microfluidic emulsification produces a broad spectrum of microgel sizes with minimal variability. The size of the microgels can be precisely controlled by adjusting the flow rate of either the oil or aqueous phase. Furthermore, productivity can be significantly increased by stacking multiple microfluidic chips or integrating several channels.^151^ Regarding the microstructural characteristics of materials, the intrinsic properties of a material-such as conductivity, resistance, flexibility, hydrophobicity, or hydrophilicity-can be more effectively harnessed in microfluidic devices due to the possibility of achieving larger interfaces between materials and fluids. Furthermore, both existing and emerging microfluidics-assisted platforms facilitate the fabrication of objects and materials with enhanced control over critical variables, including temperature, concentration and concentration gradients, flow rates, flow profiles, and mixing parameters.^27^

To date, microfluidics has been effectively employed in the fabrication of drug carriers and lubricants intended for intra-articular injection. In a study by Han et al., Nanofat was immobilized within microfluidic-generated aldehyde-modified PLGA porous microspheres (PMs) through Schiff base condensation and non-covalent binding, forming a 3D porous network (PMs@NF). These PMs significantly improved the cartilage-targeted retention efficiency of Nanofat, leading to enhanced lubrication performance and the stimulation of cytokine secretion by Nanofat-derived stem cells.^152^ Furthermore, Yang et al. successfully fabricated a monodisperse, size-uniform microsphere, specifically a poly(dopamine methacrylamide-to-sulfobetaine methacrylate)-grafted microfluidic gelatin methacrylate sphere (MGS@DMA-SBMA), utilizing microfluidic technology.^153^ This microsphere can synergize with stem cells to enhance therapeutic efficacy. In a related study, Li et al. developed a living and injectable porous hydrogel microsphere with sustained paracrine activity by employing freeze-drying microfluidic technology and incorporating platelet-derived growth factor-BB (PDGF-BB) along with exogenous mesenchymal stem cells (MSCs). The porous architecture and exceptional mechanical properties facilitate the adhesion and proliferation of exogenous stem cells, thereby mitigating the progression of OA by enhancing the supportive microenvironment and leveraging the synergistic interaction between exogenous and endogenous MSCs (Fig. 7c).^154^ In addition to hydrogels, particulate materials can be employed in the design of drug delivery systems. A drug delivery particle, decorated with 2-methacryloyloxyethyl phosphorylcholine (MPC) and composed of methacrylate anhydride-hyaluronic acid (HAMA), was developed using microfluidic electrospray technology. Leveraging the precise regulation of microfluidic electrospray flows, the resultant drug delivery particles exhibit well-defined sizes and enhanced dispersion, thereby augmenting the therapeutic efficacy for OA both in vivo and in vitro.^155^ To achieve more controlled drug release, microfluidics can also be utilized to encapsulate stimuli-responsive nanoparticles. When produced through a combination of microfluidic electrospray and cryogelation processes, the magnetic NPs effectively capture stem cell-derived exosomes, mitigating OA symptoms with the assistance of diclofenac sodium.^86^ Besides, Hou et al. developed a novel hydrogel microsphere with enhanced lubrication and drug-loading capabilities by employing radical polymerization of sulfobetaine (SB)-modified hyaluronic acid methacrylate via microfluidic technology. This biomaterial is characterized by a substantial presence of SB and carboxyl groups, which facilitate significant lubrication through hydration and enable electrostatic interactions with metformin (Met@SBHA), thereby achieving a high drug-loading capacity for the purpose of mitigating chondrocyte senescence.^156^ Furthermore, the preparation of PPKHF-loaded microfluidic hyaluronic acid methacrylate spheres, followed by their injection into the joint cavity using microfluidic technology, facilitates the targeted release of encapsulated positively charged PPKHF into the deep cartilage via electromagnetic forces, thereby enabling visualization of the drug’s location.^157^ Beyond drug delivery, the droplet generation capability of microfluidics and the mild cryogelation procedure also allow for the intelligent transport of stem cells, liposomes, and peptides.^96,158,159^ The use of microfluidic system safeguard the activity of bioactive substances.

### AI driven material design and fabrication for OA treatment

Over the past two decades, the application of ML and AI methodologies in materials design has garnered increasing interest. This trend is largely attributed to significant advancements in deep learning algorithms and the proliferation of big data generated by extensive experimental and computational pipelines.

In the biomedical domain, the objective of AI-assisted material design is not to universally surpass traditional materials across all performance metrics. Rather, it utilizes extensive datasets and sophisticated algorithms to expedite the screening of potential lead compound as well as drug repurposing, thereby enhancing design efficiency and optimizing the research and development process.^160^ Through performance predictions and structural optimizations conducted within a virtual environment, AI technology can preemptively identify and exclude unsuitable candidates, significantly reducing experimental costs and associated risks.^161^ Although this methodology is in its nascent stages, it offers researchers a more systematic and precise framework for exploration. It is anticipated to propel the advancement of novel materials and innovative applications, ultimately facilitating significant breakthroughs in the biomedical field.

Consequently, data-driven or ML-based rational material design has emerged as a prominent trend in the field of nanomedicine.^162^ Three principal methodologies for expediting the discovery of novel materials through AI techniques include: modeling the quantitative structure-property relationship (QSPR) of existing materials, optimizing the mechanical properties of current materials to fabricate novel ones, and the de novo design of novel materials through systematic modeling and nature-inspired learning.^163^

A significant challenge in OA therapy involves the development of a disease-modifying delivery system capable of controlled drug release to chondrocytes within the joint’s unique microenvironment. ML models offer detailed insights into how variations in critical material parameters influence the overall mechanical and biological behavior of the delivery system. In this context, QSPR models are frequently utilized to address formulation design challenges, aiming to optimize performance. Elgendy et al. developed an intra-articular delivery system utilizing atorvastatin-loaded lecithin-coated zein NPs (LCZN) within a thermogel matrix for the treatment of osteoarthritis. The entrapment efficiency of atorvastatin was optimized through linear regression analysis and response surface methodology, with further validation conducted via docking studies and molecular dynamics simulations. The optimized LCZN thermogel formulation demonstrated satisfactory release characteristics and anti-inflammatory properties, and it significantly attenuated articular cartilage degeneration and sub-synovial inflammatory infiltration.^164^

The application of AI in OA therapy extends to the design of functional materials with intricate three-dimensional structures. Recent studies have underscored the employment of diverse ML algorithms for data analysis, prediction, and optimization within the realm of tissue engineering.^165^ AI-assisted experimental design and data analysis confer several advantages, including the conservation of time and resources, as well as the ability to predict unforeseen optimal formulations. For instance, Patricia et al. utilized AI tools to optimize intra-articular formulations, leveraging experimental data derived from a wide array of hydrogels. The formulation presented exhibited enhanced rheological properties and markedly decreased the secretion of degradative (MMP-13) and pro-inflammatory (CXCL8) molecules.^166^ Jeong and her colleagues employed an unsupervised ANN to elucidate the relationships between hydrogel formulation and biological outcomes, identifying HA molecular weight and PEG concentration as critical parameters in the design of composite hydrogels.^167^

In addition to optimizing existing composite materials, the integration of AI and engineering technology introduces novel concepts in osteoarthritis therapy. Okesola and colleagues utilized molecular dynamics simulations to design functional coassembling hydrogels. They fabricated organic-inorganic PAH3-Lap hydrogels, which demonstrated satisfactory self-recovery properties and the capability for in situ biomineralization, thereby enhancing cell proliferation and extracellular matrix production.^168^ Furthermore, an AI-driven medical microrobotic system was developed and constructed to facilitate the in vivo repair of cartilage defects. This magnetically actuated microrobot, characterized by its porous structure, offers an innovative approach for the targeted delivery of MSCs to damaged cartilage, thereby indicating the potential for precision therapy in tissue regeneration.^169^ Furthermore, the integration of AI and advanced synthesis technologies presents a promising avenue for the treatment of osteoarthritis. Several researchers have suggested that the combination of AI, multidimensional printing and biomaterials holds significant potential to transform the therapeutic landscape for complex bone defects.^170^

While successful instances of AI-assisted design of novel scaffolds are relatively uncommon, it is anticipated that ongoing innovative efforts will increase in tandem with the revolutionary advancements in AI technology. A recent review explored the potential of engineered RNA-based and cell-based therapies for future osteoarthritis treatment. The integration of advanced cell manipulation and synthetic biology techniques with AI-driven personalized medicine may herald the advent of next-generation ‘intelligent’ cell therapies.^171^

## Perspective of material design by the next generation of AI

In the past five years, emerging techniques including generative AI and large language models have gradually demonstrated their potential in scientific research and industrial transformation. Generative AI represents a class of algorithms that facilitate the creating of new content similar to the input datasets, enabling the generation of novel material in an unexpected way. Large-scale models, leveraging big data and deep learning techniques, process and analyze vast amounts of material data to predict material properties and behaviors. Generative AI demonstrates breakthrough advantages in material development that distinguish it from traditional AI, with its core strength lying in the deep integration of creative design and systematic optimization. Using algorithms such as Generative Adversarial Networks (GANs), generative AI can directly generate novel scaffolds beyond the scope of existing knowledge, rather than merely analyzing and predicting existing data. It also leverages synthetic data to fill experimental gaps through simulating complex behaviors of materials in dynamic physiological environments. For complex multi-objective optimization scenarios, generative AI can collaboratively balance multiple constraints such as mechanical strength, biocompatibility, and degradation rate to achieve precise cross-scale design. This capability extends further into the field of personalized medicine by combining high-throughput virtual screening and 3D printing technologies, it can quickly customize topological structures that match the bone defect morphology of individual patients, significantly shortening the R&D cycle. More importantly, generative AI can integrate multi-disciplinary data, including material genomics and clinical imaging, driving the evolution of materials from “static carriers” to “self-sensing therapeutic systems”. Specifically, the application of Generative Pre-trained Transformer (GPT) models in the design of MOFs has resulted in improved accuracy in generating linker structures compared to their base model.^172^ Moreover, the advancement of ChatMOF has further diminished the learning curve associated with utilizing GPT for MOF design, highlighting the transformative potential of large language models (LLMs) when integrated with databases and ML within the domain of materials science.^173^ These advanced computational technologies empower scientists to design new materials with specific attributes from scratch, significantly advancing the field of materials science.^174^

### Key technologies and workflow for material design by generative AI

GANs enable the creation of high-quality, innovative material structures through a dual-network system consisting of a generator and a discriminator.^175^ The generator is responsible for producing novel material designs, while the discriminator evaluates their authenticity. Through iterative adversarial training, the generator progressively improves its capacity to generate realistic and functional material structures. This methodology holds significant promise for the development of novel drug carriers and biomaterials with specific application attributes. Researchers exploit the adversarial training mechanism between the generator and discriminator within GANs to design a highly sensitive and selective biosensor material. This material is capable of effectively detecting OA-related biomarkers, such as C-reactive protein (CRP) and interleukin-6 (IL-6), thereby providing a reliable assay for early OA diagnosis. By enabling rapid and accurate detection in joint fluid, this biosensor material facilitates timely clinical intervention, consequently enhancing patient outcomes.

Variational Autoencoders (VAEs) represent a crucial advancement in the field of AI-driven material design. VAEs function by encoding input data into a latent space via an encoder, subsequently reconstructing new data points from this space through a decoder.^176^ This mechanism facilitates the exploration of novel material structures by enabling manipulation within the latent space. For example, VAEs can be utilized to design polymers with specific mechanical properties by encoding existing polymer structures and generating new variants that satisfy predetermined criteria, thereby expediting the discovery of materials with unique property combinations. In the domain of tissue engineering, VAEs aid in the design of biomimetic three-dimensional scaffold materials. By leveraging the encoder and decoder of VAEs, researchers can explore and optimize various scaffold designs within the latent space, effectively mimicking the structure of natural ECM. These biomimetic 3D scaffold materials enhance chondrocyte adhesion, growth, and differentiation, providing a promising tissue engineering treatment for OA patients. This technology holds the potential to significantly accelerate cartilage tissue regeneration and repair, thereby restoring joint function and alleviating patient pain.

**Fig. 8:**
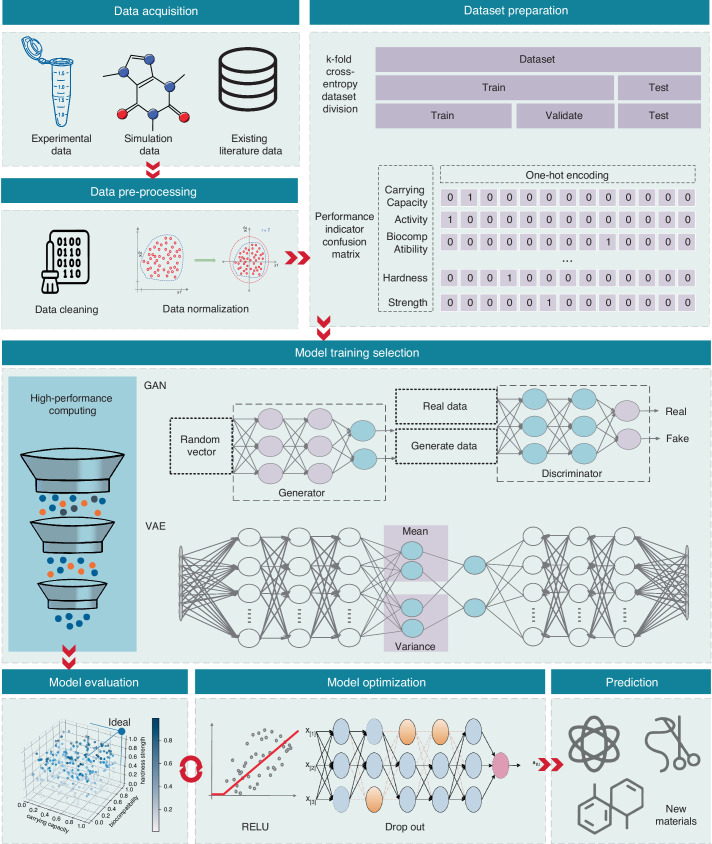
A simplified workflow for new AI-based material design. Firstly, the necessary data for modeling and model evaluation is collected, including experimental data, simulation data, and existing literature data. Then, the collected data is preprocessed through the steps of data cleaning and normalization. Next, the data set is divided into training sets, verification sets, and test sets using k-fold cross-entropy validation. And the indexes of new material performance evaluation are summarized into a confusion matrix by one-hot encoding. Secondly, the appropriate model algorithm is selected for training according to the research problem, and HPC is used if necessary. Evaluate the performance of the model using established criteria and iteratively adjust the model parameters and use techniques to optimize performance under the guidance of these standards. Finally, the forecast results are discussed and analyzed reasonably

High-performance computing (HPC) is essential for the practical implementation of large-scale AI models in the field of material design. By utilizing Graphics Processing Units (GPUs) and Tensor Processing Units (TPUs), researchers can markedly expedite the training and simulation processes of intricate models. These computational resources facilitate the handling of extensive datasets and complex simulations, thereby enabling the exploration and optimization of materials on an unprecedented scale. For instance, HPC can simulate molecular interactions of novel drug molecules, allowing for the prediction of their performance and the optimization of their structures to achieve optimal outcomes. Furthermore, HPC and large-scale models are instrumental in optimizing the structures of nanodrug carriers. These models efficiently and rapidly identify optimal carrier designs by simulating the effects of various nanostructures on drug targeting and release efficiency. Optimized nanodrug carriers enhance drug targeting and controlled release, significantly reducing systemic side effects and improving therapeutic efficacy. Their application in OA treatment will highlight the role of AI-assisted material design in enhancing drug efficacy (Fig. 8).

### Emerging reinformancement learning techniques for material design

With the advancements in data science and computational capabilities, emerging ML methodologies, particularly deep learning and reinforcement learning, are significantly transforming the field of material design.^177^ Reinforcement learning, by simulating continuous decision-making processes, demonstrates considerable potential in material design scenarios that necessitate iterative optimization. For instance, when synthesizing high-strength drug carrier materials, reinforcement learning algorithms can autonomously adjust synthesis parameters-such as temperature, pressure, and reaction time-to efficiently identify optimal performance combinations.^178^ Graph Neural Networks (GNNs), which are specialized neural networks designed for graph-structured data, excel in predicting molecular properties and optimizing drug design strategies.^179^ By representing molecules as graphs, where atoms are depicted as nodes and chemical bonds as edges, GNNs effectively capture complex intra-molecular relationships, thereby providing novel insights into drug design.

### Virtual reality technology to accelerate material design

Looking forward, emerging technologies such as Augmented Reality (AR), Virtual Reality (VR), and quantum computing are poised to significantly extend the boundaries of material design. AR and VR technologies offer immersive experiences that are transforming and revolutionizing the fields of materials science education and research. Within virtual laboratories, researchers have the capability to simulate material manufacturing and testing processes, allowing for the observation of material behaviors under diverse conditions.^180^ For example, VR technology facilitates students’ experiential learning by enabling them to engage with the reaction processes of drug carrier materials in various environments, thereby enhancing their comprehension of the materials’ properties.

Furthermore, quantum computing holds the potential to transform material design by addressing complex problems that are beyond the capabilities of classical computers. By harnessing quantum mechanical principles for extensive parallel computation, quantum computers can expedite the simulation and optimization of molecular structures. In the context of drug development, for instance, quantum computers can accurately simulate molecular interactions with target proteins, swiftly identifying promising candidates. When combined with robotic automation, the synthesis of AuNPs can be significantly improved through real-time spectroscopic feedback and ML algorithms. This integration is anticipated to facilitate the discovery of functional materials with enhanced performance by navigating a vast chemical space using intelligent search algorithms.

## Conclusion

Significant advancements have been achieved in the diagnosis and treatment of OA. Regarding diagnosis, the integration of bio-nanomaterials and medical imaging technologies has enhanced both the precision and promptness of diagnostic processes. In the realm of treatment, the development of innovative therapies, including novel biological agents, hydrogel microsphere technology, and mesenchymal stem cells, has offered patients more effective therapeutic options. Concurrently, the efficacy and safety of surgical interventions have been substantially improved. AI and ML algorithms have demonstrated exceptional capabilities in addressing factors such as shape, synthesis procedures, chemical composition, size distribution, surface modifications, self-assembly behavior, and drug loading efficiency.^181^ Notably, AI technologies not only optimize nanoparticle designs but also predict the self-assembly behavior of more complex structures, such as hydrogels. By utilizing large-scale peptide libraries and QSPR methods, researchers are poised to develop high-precision ML algorithms to guide material design, thereby further expanding the application boundaries of AI in materials science. These AI-assisted materials herald a future of more advanced and effective solutions.

## Supplementary information

Supplementary Information

Supplementary Information
