# Supplementary material for: Intelligent microstructure materials for diagnosis and treatment of osteoarthritis: progress and AI-enpowered future
Source: Bone Res. 2025 Oct 15;13:85. doi: 10.1038/s41413-025-00458-5 (PMC12528451; doi:10.1038/s41413-025-00458-5)
Supplement: Supplementary file 2 — Supplementary Information [file 41413_2025_458_MOESM2_ESM.docx]

**Supplementary Figure 1.** Schematic illustration of the evolution of the development of smart materials and its application in OA.
